# Mass media coverage and vaccination uptake: evidence from the demand for meningococcal vaccinations in Hungary

**DOI:** 10.1007/s10198-021-01296-y

**Published:** 2021-04-09

**Authors:** Anikó Bíró, Ágnes Szabó-Morvai

**Affiliations:** 1 Centre for Economic and Regional Studies, Lendület Health and Population Research Group, Tóth Kálmán utca 4, Budapest, 1097 Hungary; 2 University of Debrecen, Egyetem tér 1, Debrecen, 4032 Hungary

**Keywords:** Vaccination, Meningitis, Mass media, Imperfect information, Bounded rationality, I12, I18

## Abstract

We estimate the effect of mass media coverage of the meningococcal disease on the uptake of meningococcal vaccinations in Hungary. Our analysis is based on administrative county-level data on vaccination purchases linked to indicators of media coverage of the meningococcal disease and to administrative records of disease incidence. Using geographical and time variations in these indicators, our fixed effects estimates indicate a strong positive effect of mass media coverage of the disease on the rate of vaccination with all types of the meningococcal vaccine. At the same time, we do not find evidence that disease incidence itself has a positive impact on vaccination. These findings are broadly in line with imperfect information and the principles of bounded rationality and highlight the responsibility of mass media in influencing health-related behaviours.

## Introduction

The WHO ranked vaccination hesitancy, which is defined as “the reluctance or refusal to vaccinate despite the availability of vaccines”, among the ten most serious threats to global health in 2019 [1]. In the past decade, serious diseases such as measles have returned to developed countries, which is the suspected result of the anti-vaccine movement. A recent example is the 2014 measles outbreak in the US [2]. Vaccination plays a key role in fighting vaccine-preventable diseases for several reasons. Vaccines prevent diseases that could otherwise cause serious health problems, permanent disability or even death [3]. Vaccination is one of the most successful and cost-effective public health interventions [4]. By reducing the need to use broad-spectrum antibiotics, vaccination is important for addressing antibiotic resistance, which is also one of the ten threats on the 2019 WHO list [1]. Last but not least, vaccination promises to be the lasting solution against the COVID-19 pandemic.

Recently, the role of social media has been suspected as an important determinant of the spread of anti-vaccination sentiments ([5–7], among many others), although causal effects are difficult to establish. Betsch et al. [8] show evidence for the strong influence of vaccine-critical websites on the intentions to vaccinate. On the other hand, little is known about the effect of mass media on vaccination uptake. Smith et al. [9] find little evidence for the influence of mainstream media on MMR (measles, mumps, rubella) immunisation in the US.

There is evidence in the literature that media information about potential health hazards affects food purchases [10–12]. While that line of the literature focuses on the impact of genuinely new information (e.g., information on bird flu or mad cow disease), our study focuses on the media coverage of the meningococcal disease in general, and how that relates to vaccination demand.

We aim to extend the knowledge on the determinants of vaccination demand, focusing on the influence of mass media on vaccination uptake. Our focus is not on the refusal of mandatory vaccinations (coverage with mandatory vaccinations is almost 100% in Hungary) but on the uptake of elective vaccinations. Specifically, we examine the incidence of invasive meningococcal disease (IMD), the related media coverage, and subsequent vaccination uptake. We focus on this type of vaccination for three reasons. First, this is one of the few elective vaccinations in Hungary, which have to be purchased in pharmacies and, therefore, appear in the available administrative records. Second, IMD is a rare disease with rapid progression and a very high case-fatality ratio. As such, the effect of news is more likely identifiable compared to more common and less serious types of disease. Third, the incidence of IMD can be taken as random (see, e.g., [13, 14]), as can the related news. As a result, any change in the demand for meningococcal vaccinations after the occurrence of IMD cases or the release of related articles can be interpreted as a causal effect. Nevertheless, we apply fixed-effects models that do not build on this strong assumption.

We make use of geographic (county level) and time variations in meningitis-related news, disease incidence and vaccination rate. This research design allows us to investigate how media coverage of a disease influences vaccination demand. There is a lack of evidence on the determinants of optional vaccination demand in Hungary. The only exception we are aware of is Marek et al. [15], who document low awareness of human papillomavirus (HPV) vaccination among adolescents in Hungary.

The studies most closely related to ours are Oster [16] and Schaller et al. [17], which show evidence that pertussis (whooping cough) outbreaks increase the vaccination rate of children. While their focus is on the effect of disease outbreaks on vaccination, our focus is on the role of mass media on vaccination demand.

## Background

Meningococcal disease is caused by bacteria,^1^ which occasionally spread through the body and cause meningococcal infection. The bacteria are transmitted from person-to-person,^2^ but only very rarely become invasive, causing serious health probems [18]. Invasive meningococcal disease (IMD) often has a rapid progression, with an 8-15% case-fatality ratio. The incidence rate is the highest among young children, with a second disease peak among adolescents and young adults [19]. Meningitis and sepsis are the major clinical features of IMD, meningitis is usually caused by infection with meningococcus. IMD is notifiable and under surveillance in EU/EEA countries. There are 12 types (serogroups) of the bacteria identified, and serogroups A, B, C, X, W, and Y are responsible for the majority of the disease cases. Serogroups B and C are the most common causes of IMD in Europe, although serogroup distribution varies by location and time. Vaccines are available for the primary prevention of disease caused by serogroups A, B, C, W, and Y [18–21].

In Hungary, some vaccinations are mandatory,^3^ and these vaccinations are provided free of charge. The rate of coverage with the mandatory vaccinations is almost 100% in Hungary, higher than in the majority of other developed countries [22]. Other vaccinations, such as the meningococcal vaccines Men C, Men B, and Men ACWY are optional. If someone requires an optional vaccination, then she/he has to request a prescription from the general practitioner (GP) and purchase the vaccination at a pharmacy, which then can be administered by the GP. Since meningococcal vaccinations are optional, in principle, GPs do not instruct their patients whether they should receive the vaccination or not; they only provide information about the available vaccinations, their costs, benefits, and possible side effects. The parents decide on optional vaccinations until the child reaches the age of majority (age 18).

Three main types of vaccines are available against meningococcal disease at very different out-of-pocket prices, which are effective against different types of the disease. The Men C vaccination is partly subsidised by social security and the out-of pocket cost is approximately 300 HUF (with 1 EUR $\approx$ 300 HUF). The costs of the Men B and Men ACWY vaccinations have to be covered entirely out-of-pocket.^4^ The Men C, Men B, and Men ACWY vaccinations have been available since 2006, 2014, and 2010, respectively. Depending on at what age the immunisation is started, the Men C vaccination is given in either one or two shots (two shots are needed under the age of one), the Men B vaccination is given in either two or three shots (three shots are needed under the age of 6 months), and the Men ACWY vaccination is given in one shot. Immunisation can be started as early as the age of 2 months.[23, 24].

The reported IMD cases varied in number between 33 and 70 per year between 2006-2017 in Hungary, with 3-12 deaths per year. Considering that the total population of Hungary is approximately 10 million, IMD is a rare disease. However, the average lethality rate is very high at 15% (Table 1). Serogroup B was responsible for the majority of the IMD cases, while serogroup C was dominant only for short periods. The age-specific morbidity rate is 9.8 per 100,000 children under the age of 1 and 2.7 per 100,000 children aged 1–4 years, while the national average is 0.4 per 100,000 inhabitants [25]. As IMD is a rare disease, universal vaccination using the more costly meningococcal vaccinations, specifically the vaccination against serogroup B has been shown not to be cost-effective in other European countries [26–28].

**Table 1:** Number of IMD cases and deaths per year. (sources: [29, 30])

	2006	2007	2008	2009	2010	2011	2012	2013	2014	2015	2016	2017
IMD cases	35	49	34	39	41	70	56	54	33	36	49	41
IMD deaths	7	9	7	5	4	12	6	9	3	9	9	6

To sum up, patients face a choice between three (non-exclusive) alternatives when it comes to menningococcal vaccination. Men B is relatively expensive, but most probably provides immunity against the majority of the IMD cases in Hungary. Men ACWY is similarly costly, and protects against four types of the bacteria, which are less common in Europe (although there has been an increase of serogroup W in the past few years [19]). Alternatively, one can choose Men C as a very cheap vaccination, which protects against a serogroup which is responsible for a lower fraction of the IMD cases than serogroup B. We include all three vaccination types in our analysis to demonstrate the heterogeneity of vaccine demand for varying cost and effectiveness.

## Theoretical considerations

Demand for vaccination can be modelled as a comparison of the benefits (*B*) and costs (*C*) related to the vaccination. The benefits originate from the avoidance of the disease, and the costs are the vaccination fee and non-monetary costs such as side-effects and time costs (for formal models of vaccination demand, see [16, 31], among others). The perceived benefits equal the perceived probability of the disease ($\pi$) times the health costs of the disease (*H*). An individual opts for vaccination if

1 $$\begin{aligned} B\ge & {} C, \nonumber \\ B\;=\; & {} \pi \times H. \end{aligned}$$

If individuals are fully informed and rational, then neither the number of IMD cases nor their media coverage should affect the vaccination decision unless the disease occurs in the small neighbourhood of the decision-maker. The reason is that IMD is a very rare disease and its occurrence somewhere has no effect on the probability of re-occurrence elsewhere. Elias et al. [13] and Hoebe et al. [14] both report that the spatial or temporal clustering of IMD cases beyond chance is rare in both Germany and the Netherlands.

There is plenty of evidence in the literature that the media influence people’s behaviour (for a thorough review see [32] as well as [33]), including fertility and divorce decisions [34, 35] and even violence [36]. In recent years, a growing body of evidence has emerged, which suggests that social media also exert a significant effect on vaccination decisions, too ([5–7], among others).

There are two channels that can mediate the effect of media on behaviour. First, if individuals are rational but not fully informed, then media news may provide relevant information for them [37–39]. In this case, the media can exert an effect even if the information is biased and the readers are aware of that, i.e., if the medium prefers shocking news [40–42]. If individuals are rational but not fully informed then learning about the details of an IMD case or a death due to IMD might impact the perceived severity of the disease (*H*), thus potentially increasing the observed benefits of the vaccination. The individuals can achieve such additional information by the media coverage of IMD.

Second, the news may have a direct behavioural effect in case of bounded rationality. For instance, this might be the case if individuals are myopic, that is, if they pay attention to recent cases [16], or if they overestimate and overweight small probabilities [43]. In the case of bounded rationality and imperfect information, media coverage of IMD might further affect vaccination demand. When faced with decisions under uncertainty (such as the decision on vaccination), people rely on heuristic principles to simplify the complex tasks of assessing likelihoods and predicting values [44]. One such heuristic is availability, i.e., people assess the probability of an event by the ease at which occurrences of the event could be brought to mind [44, 45].

Tsutsui et al. [46] apply a model of bounded rationality to analyse the demand for influenza vaccination. They conclude that the dissemination of information about the vaccination is especially important among people who are inexperienced with the vaccination. As Sadique et al. [47] note, the severity of the health effects associated with both diseases and vaccination-associated adverse events exerts an important influence on the demand for vaccination.

Betsch et al. [8] and Chen and Stevens [48] argue that the vividness and salience of case-based information related to vaccination risks can lead to vaccination refusal. We argue that the salience of a vaccine-preventable disease (such as IMD) can, on the other hand, lead to a higher demand for vaccination. This argument is in line with the availability hypothesis, i.e., people perceive the infection probability ($\pi$) to be higher if they can recall IMD cases due to related media coverage. In addition, Kahneman and Tversky [49] argue that very low probabilities are generally overweighted, possibly contributing to the attractiveness of vaccination in the case of rare diseases, such as IMD. Additionally, there is evidence in the literature that events happening in closer proximity have bigger effect on behaviours [50, 51].

An additional policy-relevant issue is which subpopulations are more responsive to vaccination-related news of the media. Ackerberg [52] and Tellis et al. [53] find that less-informed individuals are more responsive to additional information, such as advertisements. The level of education might also matter, as higher-educated individuals read news more often than those who are less educated (see, e.g., [54–56]). In line with that, Qian et al. [57] finds that higher-educated mothers react with a stronger behaviour change to the claim that the MMR vaccine causes autism.

Theoretically, it is not obvious, how long the impact of the media on vaccination uptake may last. We might see the impacts to persist over a few weeks because patients (or their parents) wishing to receive the vaccination have to contact the GP both for prescription and for the administration of the vaccination. It is possible that in case of vaccinations administered mainly to infants (Men B and C), the effect lasts for more periods, as parents of newborns need to wait until 2 months of age to vaccinate. Also, there might be permanent effects if the perceived costs and benefits of immunization change due to an extensive, albeit transitory media coverage. Following the availability hypothesis, the impact is expected to decrease over time once the media stop focusing on the disease. The related literature suggest only temporary impact of media coverage on health-related behaviours (see, e.g., [10, 58]).

## Data sources

Aggregated vaccination statistics were provided by the National Health Insurance Fund Management (NHIF). These are monthly, county-level statistics on the number of meningococcal vaccination purchases at pharmacies, out-of-pocket spending, and social security spending. The time coverage is January 2009–December 2018, and the database includes all vaccination purchases made within this period. Location (county) corresponds to the location of the pharmacy where the purchase occurred. Because the Men B vaccination became available only in 2014, in the main analysis, we restrict the sample to 2014–2018 to have the same sample period for all three types of vaccinations. In the Appendix, we present the results for the entire available data period. In addition to the data on meningococcal vaccination, for the sake of a placebo analysis, we also use monthly county-level statistics on vaccination against tick-borne encephalitis, which also originates from the NHIF and covers the same time span.

As a supplementary data source, we utilise individual-level vaccination data provided by the National Healthcare Services Center (NHSC).^5^ This dataset includes all children who were born between 2008 and 2017 and who were recorded to have an outpatient or inpatient event or a receipt of a state-subsidised medication. The data are linked with the birth registry of the Hungarian Central Statistical Office (HCSO), based on the place and date of birth of the children. After keeping only exact matches, the linkage ratio is 54 percent. The linkage ratio is lower in urban areas. As a result, this database has three important limitations. First, due to imperfect matching, the observed sample is not representative of the entire population. Second, only state-subsidized Men C vaccinations are recorded, while the non-subsidized types B and ACWY are not recorded. Third, we have to constrain our sample to 0- to 2-year-olds to avoid censoring problems (3-year-olds are observed only from 2011, 4-year-olds from 2012, and so on). We also exclude year 2017 to avoid the problem that we might not observe the immunisation of some newborns of year 2017. Due to the imperfect and non-representative matching and age restriction, in the linked C-HCSO (individual level) data, we observe 38% of the Men C vaccinations reported by the NHIF (county level data) in years 2010–2016. In turn, the linked NHSC-HCSO data include individual characteristics, such as the level of education of the mother and father and the type of municipality of living, which provide further insights into the heterogeneity of vaccination uptake.

County-specific monthly numbers of cases of IMD and annual county-specific IMD death numbers were provided by the National Center for Epidemiology, Department of Epidemics and Infection Control. The time period of the coverage is January 2009–December 2017 for IMD cases and 2010–2018 for IMD deaths. In the main analysis, we restrict the observations to 2014–2018, and we fill up the missing number of IMD cases in 2018 with zeroes. While this implies a measurement error in the IMD cases in 2018, the regression estimation results are robust to excluding year 2018 from the analysis.

County-specific annual statistics on population size and the number of children’s general practitioners (GPs) stem from the T-STAR regional statistical database of the Hungarian Central Statistical Office. The county-specific ratio of the population having at least a secondary education level originates from the Population Census of 2011, and the statistics are reported by the Hungarian Central Statistical Office [59].

We collected statistics of the online media coverage of the meningococcal disease and vaccination using web scraping techniques and looked at the four most popular online journals of Hungary.^6^ We scraped the news contents of these journals using the keyword of meningitis (agyhártyagyulladás, in Hungarian). We scraped only the article titles; thus, if the keyword appeared only in the article’s text but not in its title, then that article was not considered in our analysis.^7^ Additionally, we restrict our attention to those articles that refer to Hungary. We calculate the monthly number of all meningitis-related articles and meningitis-related articles that referred to a death case. We also count the number of meningitis death cases reported by the media. Typically, if the media report a death case, then several articles are published related to the same case. Therefore, the number of articles related to meningococcal death cases is much higher than the number of reported death cases. The majority of the articles refer to a specific county (typically, it is reported in which county the reported illness occurred); thus, we can generate county-specific indicators of mass media coverage.

Finally, to check whether individuals’ web browsing (information searching) activity moves in line with the mass media coverage, we also look at county-specific browsing history data from Google trends, again using the keyword of meningitis (agyhártyagyulladás, in Hungarian).

## Methods

We start our empirical analysis with the provision of descriptive evidence on geographic variations in vaccination rates, and on the time patterns of and comovement between vaccination uptake, media coverage and online searching activity. Using the individual-level vaccination data (linked NHSC-HCSO data), we also provide descriptive results on the heterogeneities in vaccination uptake by parental education and living area.

We then turn to regression analyses^8^. Let $v_{mct}$ denote the number of type *m* vaccinations purchased per 100,000 inhabitants aged 0–17 in county *c* on monthly date *t*.^9^

First, to provide insights into the determinants of cross-county variation in vaccination uptake, we analyse how county-specific unemployment rate (*unemp*), the fraction of individuals with at least a secondary education (*edu*) and children’s GP availability (children’s GP per 100,000 inhabitants aged 0–17, *GP*) relate to vaccination rate:

2 $$\begin{aligned} v_{mct}=\alpha _{0}+\alpha _{1} unemp_{ct}+\alpha _{2} edu_{c}+\alpha _{3} GP_{ct}+D_t \alpha _{d}+\epsilon _{mct}, \end{aligned}$$

where $D_t$ are time (monthly date) dummies. Due to the lack of time variation in *edu* and the moderate time-variation in the variables *unemp* and *GP*, we cannot include county effects in equation (2). The *unemp* and *edu* variables serve as proxies of the county-specific average level of socio-economic status, whereas the *GP* variable serves to capture the ease of access to the vaccination.

Second, we investigate how mass media coverage and the number of IMD cases impact vaccination uptake, adding lags of $1-4$ months to the model. We normalise the number of IMD cases with the size of the population aged 0–17 ($IMD_{ct}$). The indicators of mass media coverage are also county specific. We include either the county $\times$ month-specific number of meningitis-related online articles ($article_{ct}$) or those articles that refer to a death case ($death_{ct}$). For identification, we exploit the observation from previous literature that events at a closer distance have a larger effect on human behaviour [50, 51]. In our empirical approach, we measure the response difference between counties covered by the news versus all the other counties. If a county-specific meningitis-related article affects vaccination in other counties as well, we are underestimating the total effect. The advantage of this approach is that we can control for any events that coincide in time with the media coverage and could introduce upward bias in our estimations. Thus, our estimates can be considered as lower bounds for the total effects of mass media coverage on vaccination uptake.

We estimate the following equations:

3 $$\begin{aligned} v_{mct}= \;& {} \beta _{0}+ \sum _{k=0}^{k=4} \beta _{1}^{k} article_{c,t-k}+ \sum _{k=0}^{k=4} \beta _{2}^{k} IMD_{c,t-k}+D_t \beta _{d}+ D_c \beta _{c}+\nu _{mct}, \end{aligned}$$

4 $$\begin{aligned} v_{mct}= \;& {} \tilde{\beta _{0}}+ \sum _{k=0}^{k=4} {\tilde{\beta }}_{1}^{k} death_{c,t-k}+ \sum _{k=0}^{k=4} {\tilde{\beta }}_{2}^{k} IMD_{c,t-k}+D_t {\tilde{\beta }}_{d}+ D_c {\tilde{\beta }}_{c}+{\tilde{\nu }}_{mct}. \end{aligned}$$

The $D_t$ time (monthly date) dummies capture the effects of country-wide factors, such as vaccination price changes or aggregate trends in the media coverage of the meningococcal disease. $D_c$ are county dummies, which capture such factors as the average welfare in a county and the availability of healthcare services. These county-specific factors are likely related both to the vaccination demand and the incidence of IMD, the latter of which in turn relates to the likelihood of mass media coverage of meningitis in the given county. As a specification check, we replace the incidence indicators ($IMD_{ct}$) with a mortality indicator, namely, the population weighted, county-specific annual number of IMD deaths. Additionally, to analyse the importance of time coverage, we re-estimate equation (3) over the entire observation period (2009–2018 in case of Men C, 2011–2018 in case of Men ACWY).

In equations (3) and (4), the $\beta _1$ and $\tilde{\beta _1}$ parameters show if holding the IMD incidence rate fixed, mass media coverage has an additional effect on vaccination uptake. In turn, the $\beta _2$ and $\tilde{\beta _2}$ parameters show if the IMD incidence rate affects the vaccination uptake, holding mass media coverage fixed. News about new IMD cases might spread locally even without coverage by the mass media (e.g., via social networks), thus, in principle, IMD incidence rate might have a direct effect on vaccination uptake. As only a few IMD cases are covered by the mass media, multicollinearity does not arise due to the joint inclusion in the model of the *article* and *IMD* indicators (or the *death* and *IMD* indicators)^10^. If only the mass media coverage were included in these regressions, we could not make sure that the estimates reflect the effect of news, and not the effect of IMD cases themselves.

To provide further evidence on the causal link between news and vaccination demand, we test for information collection through the internet on vaccination. Specifically, we estimate equations (5) and (6), where the outcome variable is the county $\times$ month specific indicator of Google search intensity for the term menningitis (“agyhártyagyulladás”), denoted by $g_{ct}$:

5 $$\begin{aligned} g_{ct}=\; & {} \gamma _{0}+ \sum _{k=0}^{k=4} \gamma _{1}^{k} article_{c,t-k}+ \sum _{k=0}^{k=4} \gamma _{2}^{k} IMD_{c,t-k}+D_t \gamma _{d}+ D_c \gamma _{c}+\eta _{ct}, \end{aligned}$$

6 $$\begin{aligned} g_{ct}=\; & {} \tilde{\gamma _{0}}+ \sum _{k=0}^{k=4} {\tilde{\gamma }}_{1}^{k} death_{c,t-k}+ \sum _{k=0}^{k=4} {\tilde{\gamma }}_{2}^{k} IMD_{c,t-k}+D_t {\tilde{\gamma }}_{d}+ D_c {\tilde{\gamma }}_{c}+{\tilde{\eta }}_{ct}. \end{aligned}$$

Although the dependent variable in equations (5) and (6) lies between 0 and 100, we prefer the linear specification over a fractional response model or a beta regression for the sake of the ease of interpretation of the results. Also, as we are interested in the coefficient estimates and not in predictions, predictive values possibly falling outside the 0–100 interval is less of a problem in our application.

Next, we analyse heterogeneities in the impact of meningitis-related news on vaccination uptake by three dimensions: county-specific unemployment rate, county-specific availability of children’s GP and county-specific ratio of individuals with at least a secondary education. To do so, we conduct split-sample analyses, splitting the sample at the year-specific median of the three listed heterogeneity indicators. We re-estimate equation (3) based on these subsamples. The main benefit of the split-sample analysis over adding interaction terms between the indicator of mass media coverage and the heterogeneity indicators is that it allows heterogeneities in the coefficients of all regressors, including the time and county effects.

Finally, as a placebo check, we re-estimate equation (3) with the per capita number of purchased vaccinations against tick-borne encephalitis as the outcome variable. This vaccination is also optional, with out-of-pocket costs of approximately 5300 HUF per vaccination. Again, for the sake of comparability, we calculate the number of vaccinations per 100,000 inhabitants aged 0–17. In principle, the articles related to IMD should have little effect on the uptake of vaccinations against tick-borne encephalitis because a case of IMD does not have any effect on the risk of tick-borne encephalitis. A small effect may arise due to the raising awareness of immunisation.

## Results

### Descriptive statistics

**Fig. 1:**
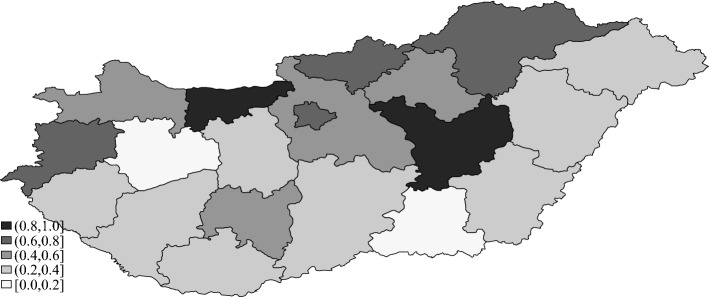
Cases of invasive meningococcal disease per year per 100,000 inhabitants (average over 2009–2017)

Figure 1 shows the regional variation in IMD incidence in Hungary. The annual number of IMD cases per 100,000 inhabitants per county varies between 0.13 and 0.81. The incidence rate is generally higher in the northern part of the country, with an average value of 0.61 in the capital city of Budapest (where one-fifth of the population of Hungary lives).

The average out-of-pocket unit costs of the three types of meningococcal vaccinations vary by vaccination type. Additionally, there are slight variations in prices between pharmacies. Men C is the cheapest vaccination, partly, because it is subsidised. The subsidy rate was 70% until the end of 2016. The subsidisation rate increased in January 2017; since then, the out-of-pocket cost is 300 HUF, but only for patients aged under 2 years (300 HUF $\approx$ 1 EUR). The Men B and Men ACWY vaccinations are much more expensive, as these are not subsidised. The unit cost of the Men B vaccination remained at approximately 30,000 HUF from its introduction in 2014 until 2018. Considering that immunisation against serogroup B requires 2 or 3 shots, and that the average monthly net salary was approximately 200,000 HUF in 2017 [60], immunisation against serogroup B costs, on average, approximately half of a parent’s monthly salary. In the period of our analysis, the price of Men ACWY gradually fell from approximately HUF 16,000 per shot to approximately HUF 11,000. Until the end of 2016, considering that the Men C vaccination had to be given in two shots, while the Men ACWY was given in only one shot, the out-of-pocket total costs of the two vaccinations were comparable (the total cost of Men C was approximately 30–50% that of Men ACWY for children under the age of 2).

**Fig. 2:**
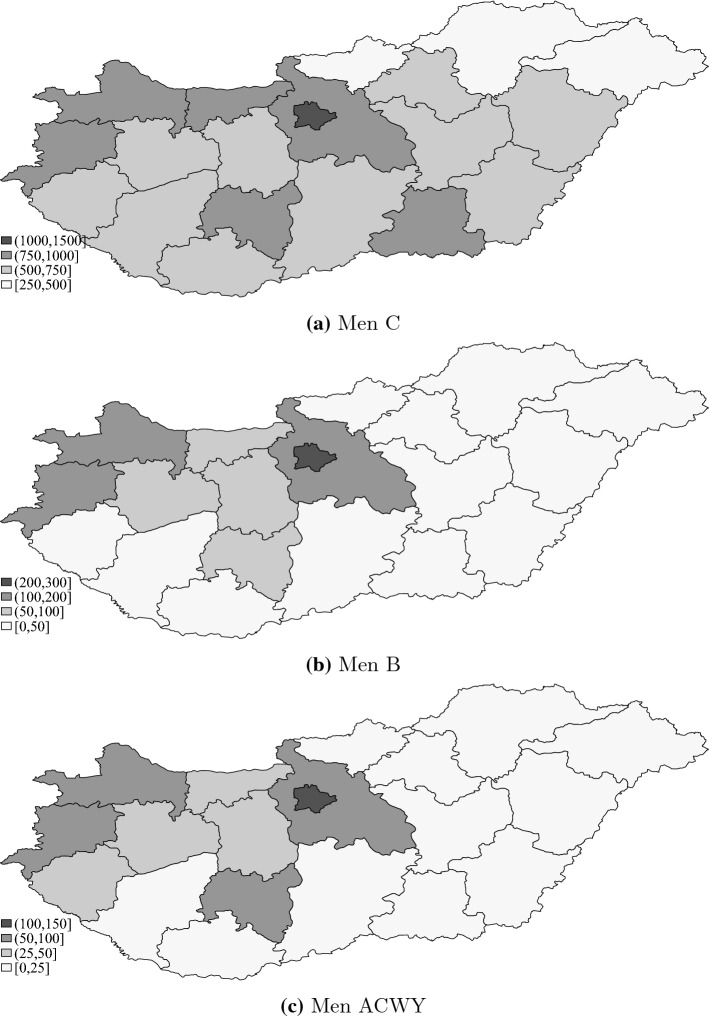
Average monthly number of meningococcal vaccinations purchased per 100,000 population aged 0–17 over years 2014–2018

The maps of Fig. 2 indicate that the per capita demand for all three types of meningococcal vaccination tends to be higher in the capital city and in the western part of the country, which is typically richer and more developed. The differences in vaccination purchases are large. The per capita number of Men C vaccinations purchased is 4 times larger in the county with the highest purchase rate than in the county with the lowest purchase rate. The same ratio is approximately 15 in the case of the more expensive vaccinations (Men B and Men ACWY).

Table 2 shows the vaccination rate at ages 0–2 by parental education and living area. We see clear evidence for a socio-economic gradient: children of more educated parents and living in urban (typically wealthier) areas are more likely to receive the Men C vaccination than are other children. For instance, a child whose mother has less than 8 years of education has a 7.9% probability of receiving the Men C vaccination, whereas a child whose mother is tertiarily educated has a 66% probability of receiving the vaccination. Additionally, while the rate of vaccination is 75% in the capital city, it is only 34.5% in villages. Further results indicate that the vaccination rate is the lowest among the children of lower-educated parents who live in villages.

**Table 2:** Socio-economic differences in Men C vaccination rate at ages 0–2

Mother’s education	Mean	95% CI	Father’s education	Mean	95% CI
0–7 years	0.079	(0.078–0.081)	0–7 years	0.097	(0.094–0.099)
8 years	0.199	(0.199–0.200)	8 years	0.241	(0.240–0.241)
Vocational school	0.401	(0.401–0.402)	Vocational school	0.455	(0.455–0.456)
Grammar school	0.555	(0.554–0.555)	Grammar school	0.587	(0.586–0.588)
Tertiary	0.659	(0.658–0.660)	Tertiary	0.673	(0.672–0.673)

Living area	Mean	95% CI			
Budapest	0.751	(0.750–0.753)			
Town	0.544	(0.544–0.545)			
Village	0.345	(0.345–0.346)			

The table shows the probability of having received one Men C vaccination until the age of 2, over years 2010–2016. The statistics are based on the linked NHSC-HCSO data (as explained in Sect. 4)

**Fig. 3:**
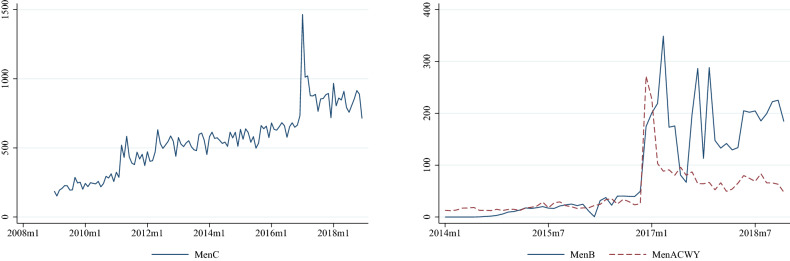
Monthly number of meningococcal vaccinations purchased per 100,000 population aged 0–17

The time trends of county-specific per capita number of vaccinations are displayed in Fig. 3. The demand for the Men C vaccination is much higher than that for the Men B and Men ACWY vaccinations. Even after 2017, when the demand for all three types of vaccinations increased, the per capita quantity of Men C purchases remained approximately 10–20 times higher than that of the other two meningococcal vaccinations. The differences in the levels of demand are in line with the price differences; i.e., the cheapest version of the meningococcal vaccinations is the most demanded.

Figure 3 also indicates that the vaccination rate jumped up in 2017 for all three types of meningococcal vaccinations. This could potentially be explained by the increased subsidisation rate of the Men C vaccination; however, that should not have such a large effect on the other two vaccination types. Instead, the jump in the demand coincides with the massive media coverage of the sudden death of a secondary school student in Budapest due to IMD in December 2016, followed by the death of a 2-year-old child in Borsod County.

**Fig. 4:**
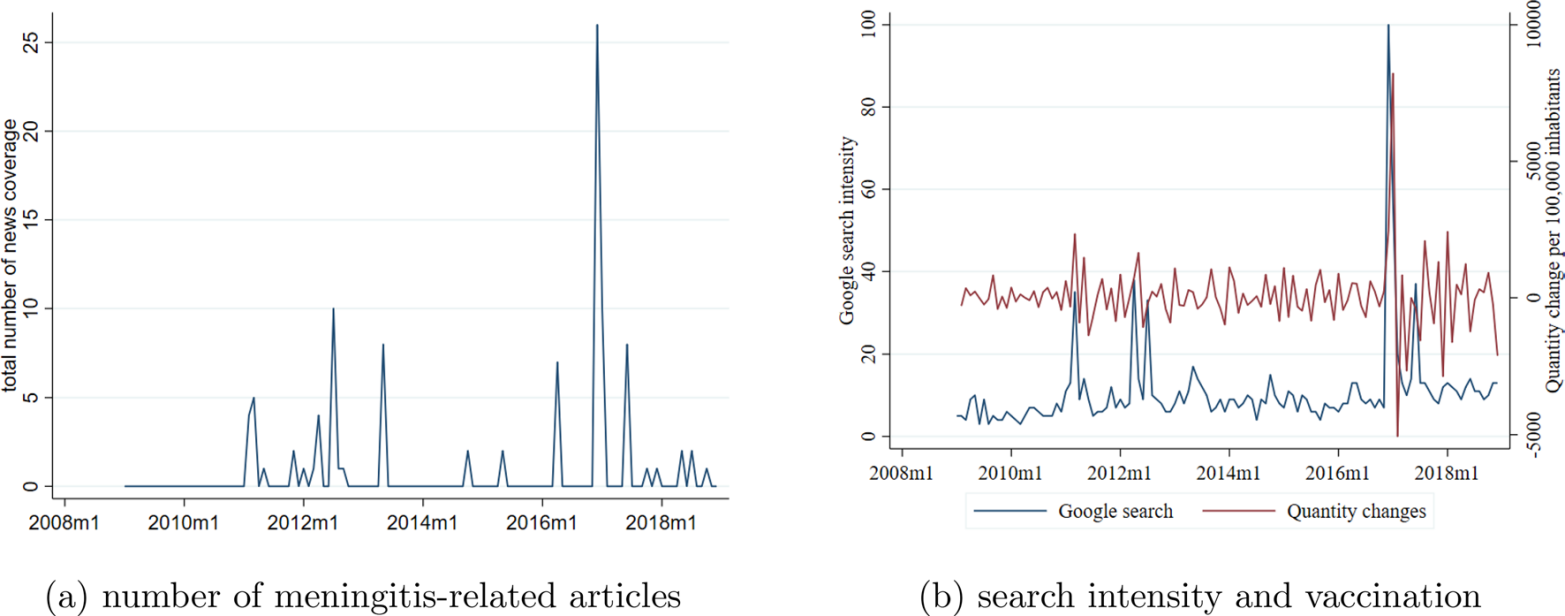
Mass media coverage, online search patterns related to meningitis and first differenced uptake of meningococcal vaccinations

The time trend in the monthly number of online newspaper articles related to meningitis is displayed in panel *a* of Fig. 4. Over 2009–2018, we found 100 meningitis-related articles (with a monthly average number of articles of 0.7), out of which 65 articles referred to a death case. Over the same time interval, 7 death cases due to IMD were covered by at least one of the analysed online journals. The peak in the number of articles in December 2016 corresponds to the two death cases mentioned above. Panel *b* of Fig. 4 indicates that information search patterns closely follow the mass media coverage of meningitis, and there is a strong co-movement between vaccination uptake and online search intensity.

**Fig. 5:**
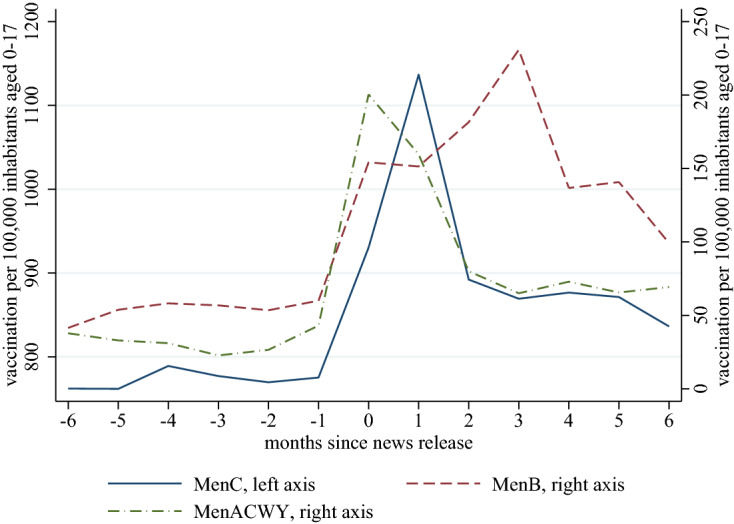
Vaccination uptake per 100,000 population aged 0–17, as function of months elapsed since a news release (years 2014–2018)

As the last piece of descriptive evidence, in Fig. 5, we provide an event-study type plot. Time=0 corresponds to the month when a meningitis-related online article was released, conditional on no article release the previous 6 months (to ensure a sufficient length of comparison period). We then plot the monthly vaccination rate as a function of the months elapsed since the news release. We see 1.5-5-fold jumps in the rates of vaccination, with more substantial relative jumps in the less-demanded vaccinations.

### Baseline results

The results of Table 3 reveal that the demand for all three types of meningococcal vaccination is higher in the more affluent counties, i.e., in counties with lower unemployment rates and with more-educated populations. This outcome is in line with the descriptive evidence reported in Table 2. The demand for the Men C vaccination also seems to be higher where the per capita number of children’s GPs is higher; thus, primary care availability matters for the vaccination rate. However, the per capita number of children’s GPs does not seem to matter for the demand for more expensive meningococcal vaccinations. It is important to note that county fixed effects are not included in these regressions; thus, the three county-specific indicators can capture many other unobserved factors (such as health status and health care attitudes of the population) and these results should not be interpreted as causal.

**Table 3:** Association between county-specific unemployment rate, children’s GP availability and vaccination rate

	Men C	Men B	Men ACWY
Unemployment rate (%)	$$-$$41.5$$***$$	$$-$$4.31$$***$$	$$-$$2.71$$***$$
	[1.38]	[0.99]	[0.55]
Fraction of at least secondary educated (%)	13.6$$***$$	7.62$$***$$	3.60$$***$$
	[0.66]	[0.90]	[0.54]
Children’s GP per 100,000 inhabitants aged 0–17	4.75$$***$$	$$-$$0.31$$*$$	$$-$$0.19$$**$$
	[0.31]	[0.17]	[0.08]
Monthly date effects	yes	yes	yes
Mean of outcome	680	72	36
Number of observations	1,200	1,080	1,200

Robust standard errors in brackets, *** *p*<0.01, ** *p*<0.05, * *p*<0.1. Sample: years 2014–2018 (from July 2014 in case of Men B). Outcome variable: vaccination per 100,000 inhabitants aged 0–17. Mean unemployment rate: 5.8%; mean fraction of at least secondary educated: 44.8%; mean number of children’s GP per 100,000 inhabitants aged 0–17: 81.6

Table 4 presents the main results. Net of monthly date effects and county effects, if a county-specific meningitis-related article is published then that has a positive effect on the uptake of meningococcal vaccinations and on online search activities. This effect declines substantially after 1–2 months, except for the Men B vaccination, where the effect is estimated to persist even 4 months after the release of the article. This absolute immediate effect is similar for the Men C and Men B vaccinations (additional 17–18 vaccinations per 100,000 people aged 0–17), although compared to the mean vaccination rate, the relative increase is larger in the uptake of the Men B vaccination. The relative effect of online media coverage of meningitis is by far the largest on the Men ACWY vaccination (an additional 41 vaccinations compared to the mean of 36 per 100,000 people aged 0–17). These effects are slightly larger if the article refers to a death case. On the other hand, we do not see evidence that actual IMD cases would statistically significantly affect vaccination uptake, although there is a weak positive effect on online search activities.

In the bottom of Table 4, we also report the sum of the estimated coefficients of current and lagged meningitis-related articles. Over a 5-month period, the cumulative effect of the news is statistically significant on all three types of meningococcal vaccination, but it is larger on the Men B and Men ACWY vaccinations than on the Men C vaccination. The findings indicate that the release of the news not only changes the timing of the vaccination (vaccinations are brought forward as a result of the news), but also leads to an increased total demand over a 5-month period.

Appendix Table 7 shows that if both county fixed effects and monthly date fixed effects are excluded from equation (3) then mass media coverage is estimated to have a stronger positive effect on the demand for all three types of meningococcal vaccination. Including county effects (to eliminate the influence of county-specific time-invariant factors) reduces the estimated effects, but these estimated effects are still higher than those obtained under the preferred baseline specifications (Table 4). This outcome suggests that if country-wide time trends in vaccination demand are not taken into account then the effect of mass media coverage on vaccination demand is overestimated.

**Table 4:** Fixed effects models of vaccination uptake and online search intensity

	All articles			Death related articles			All articles	Death related articles
	(1)	(2)	(3)	(4)	(5)	(6)	(7)	(8)
	Men C	Men B	Men ACWY	Men C	Men B	Men ACWY	Search	Search
Number of county-specific articles								
Current	17.79$$***$$	16.92$$***$$	41.23$$***$$	20.37$$***$$	18.04$$***$$	42.65$$***$$	1.644$$**$$	1.502$$***$$
	[1.627]	[2.921]	[5.855]	[1.407]	[1.914]	[4.998]	[0.601]	[0.523]
1 Month lag	21.29$$***$$	10.61$$***$$	25.93$$***$$	21.46$$***$$	12.21$$***$$	27.74$$***$$	0.446	0.435
	[2.591]	[2.299]	[2.715]	[1.953]	[1.975]	[3.177]	[0.327]	[0.322]
2 Months lag	$$-$$4.289$$**$$	14.71$$***$$	6.963$$***$$	$$-$$2.674	14.64$$***$$	7.424$$***$$	0.116	0.0626
	[1.786]	[2.728]	[1.046]	[1.851]	[2.264]	[1.420]	[0.252]	[0.225]
3 Months lag	$$-$$1.213	36.06$$***$$	6.068$$***$$	$$-$$0.313	38.42$$***$$	6.360$$***$$	0.381	0.297
	[2.268]	[8.322]	[1.472]	[2.090]	[6.630]	[1.238]	[0.028]	[0.217]
4 Months lag	$$-$$1.872	10.14$$***$$	6.411$$***$$	$$-$$3.356$$**$$	11.82$$***$$	6.024$$***$$	$$-$$0.0801	$$-$$0.160
	[1.459]	[2.533]	[0.786]	[1.384]	[2.437]	[0.832]	[0.023]	[0.187]
IMD cases per 100,000 population aged 0$-$17								
Current	$$-$$0.717	$$-$$0.0946	$$-$$0.797	$$-$$0.776	0.0815	$$-$$0.325	1.980$$**$$	2.020$$**$$
	[2.697]	[4.189]	[2.442]	[2.692]	[4.101]	[2.254]	[0.928]	[0.923]
1 Month lag	$-$1.830	0.584	$-$0.298	$-$1.156	0.715	0.937	0.161	0.222
	[2.842]	[2.179]	[1.603]	[2.835]	[2.065]	[1.367]	[0.523]	[0.512]
2 Months lag	1.631	0.431	2.117	2.411	1.028	3.559$$**$$	$$-$$0.790	$$-$$0.742
	[4.174]	[2.640]	[1.388]	[4.698]	[2.809]	[1.629]	[0.792]	[0.791]
3 Months lag	5.170	$$-$$1.265	2.283$$**$$	5.094	$$-$$0.288	2.605$$*$$	0.0230	0.0354
	[3.561]	[4.603]	[1.025]	[3.541]	[4.258]	[1.310]	[1.100]	[1.090]
4 Months lag	$$-$$0.668	0.702	1.395	$$-$$0.868	1.823	1.774$$**$$	$$-$$0.853	$$-$$0.824
	[2.289]	[2.420]	[0.872]	[2.251]	[2.100]	[0.760]	[0.849]	[0.854]
County effects	yes	yes	yes	yes	yes	yes	yes	yes
Monthly date effects	yes	yes	yes	yes	yes	yes	yes	yes
Mean of outcome	680	72	36	680	72	36	15.553	15.553
Sum of nr of article coefficients	31.71$$***$$	88.43$$***$$	86.6$$***$$	35.49$$***$$	95.13$$***$$	90.19$$***$$	2.508$$***$$	2.137$$***$$
	[4.675]	[17.86]	[11.4]	[5.007]	[14.32]	[11.38]	[1.075]	[0.745]
Number of observations	1,200	1,080	1,200	1,200	1,080	1,200	1,200	1,200

Robust standard errors in brackets, $$***$$* p* < 0.01, $$**$$ *p* < 0.05, $$*$$* p* < 0.1. Sample: years 2014$$-$$2018 (from July 2014 in case of Men B). All meningitis$$-$$related articles are considered in models (1$$-$$3) and (7). Articles referring to an IMD death case are considered in models (4$$-$$6) and (8). In models (1$$-$$6), the outcome is the monthly vaccination rate per 100,000 population aged 0$$-$$17. In models (7$$-$$8) the outcome is the Google search intensity on the 0–100 scale

The results reported in Appendix B (Appendix Fig. 7) show that extending the time coverage to the entire observation period (years 2009–2018 in the case of the Men C vaccination and 2011–2018 in the case of the Men ACWY vaccination) has little effect on the estimated relation between online media coverage and vaccination uptake, although the precision of the estimates decreases.

The estimated effects of county-specific meningitis-related news on vaccination uptake change little if instead of the IMD incidence rate, the annual mortality rate is included as a regressor (Table 5). A statistically significant and strong positive effect of media coverage is estimated on the uptake of all three types of meningococcal vaccinations. At the same time, the actual number of IMD death cases has no or even a negative (albeit small) effect on the demand for meningococcal vaccinations.

**Table 5:** Fixed effects models of vaccination uptake, annual county-specific IMD mortality rate included as regressor

	(1)	(2)	(3)
	Men C	Men B	Men ACWY
Number of county-specific articles			
Current	17.89$$***$$	17.01$$***$$	41.19$$***$$
	[1.326]	[2.726]	[5.889]
1 Month lag	21.68$$***$$	10.72$$***$$	26.09$$***$$
	[3.070]	[1.969]	[2.569]
2 Months lag	$$-$$3.419$$*$$	14.70$$***$$	7.329$$***$$
	[1.703]	[2.508]	[0.837]
3 Months lag	$$-$$0.605	36.04$$***$$	6.358$$***$$
	[19.48]	[82.09]	[12.20]
4 Months lag	$$-$$1.628	10.25$$***$$	6.568$$***$$
	[1.601]	[2.182]	[0.6900]
Annual IMD deaths per 100,000 population aged 0–17 in the county			
Current year	$$-$$7.970$$***$$	$$-$$0.939	$$-$$0.420
	[2.239]	[4.762]	[1.381]
County effects	yes	yes	yes
Monthly date effects	yes	yes	yes
Mean of outcome	680	72	36
Number of observations	1,200	1,080	1,200

Robust standard errors in brackets, $$***$$* p* < 0.01, $$**$$* p* < 0.05, $$*$$* p* < 0.1

Sample: years 2014–2018 (from July 2014 in case of Men B)

All meningitis-related articles are considered. The outcome is the monthly vaccination rate per 100,000 inhabitants aged 0–17

To sum up, the results suggest that as a response to the news, people increase their demand for the costly vaccines effective against serogroups with higher prevalence, whereas the demand is less responsive for the cheaper vaccine. For someone to be able to respond with an increased demand for the more expensive vaccines, a relatively high income is needed. Also, one has to make an informed decision, knowing how the cheap and the expensive vaccines differ. To be relatively more informed, one needs to be higher educated on average, or needs to have a discussion with a GP. We test these hypotheses in the next section.

### Heterogeneity analysis

**Fig. 6:**
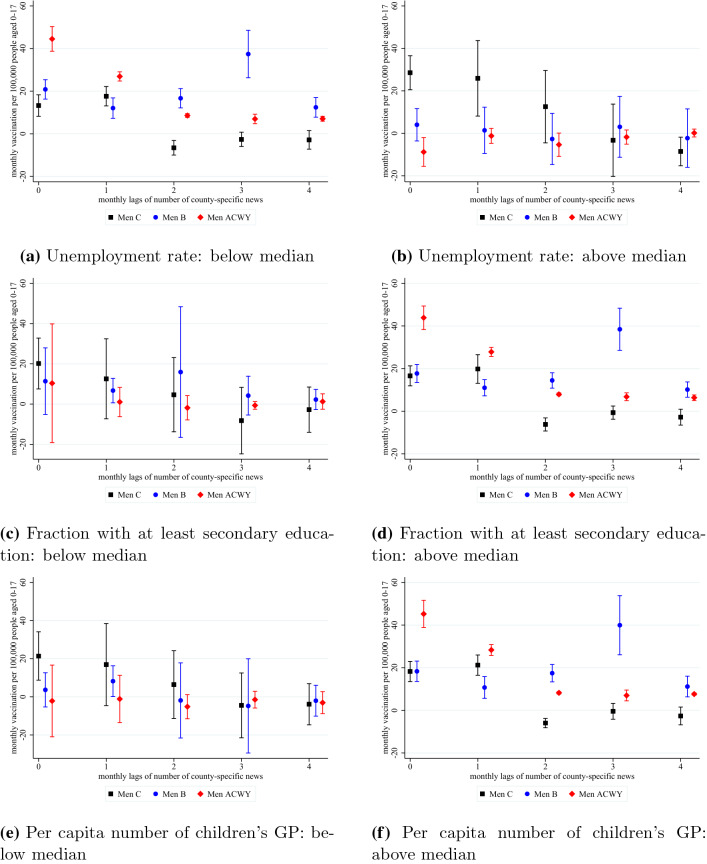
Heterogeneity in the effect of meningitis-related online articles on vaccination (with 95% CI)

Figure 6 summarises the estimation results of the heterogeneity analysis. We divide the counties based on unemployment rate (as a proxy for income), education level and children’s GP density. The plots in the first row indicate that in lower income (higher unemployment) counties, the demand for the more expensive vaccinations are not increased by the mass media coverage, however, we see a significant effect on the cheap vaccination. On the other hand, in the higher income counties (with lower unemployment rates), we find a smaller but still significant effect on the cheap vaccinations and an even higher effect on the more expensive vaccinations.

The plots in the second and the third rows of Fig. 6

indicate that in counties where education levels and GP densities are below the median, most of the effects of the mass media coverage are zero and statistically insignificant. On the other hand, in counties with a higher level of education and more GPs, the effects are more substantial. This suggests that being informed is essential for the demand effects of the news, in line with earlier results in the literature that on average, the impact of media is stronger among the more-educated individuals ( [57], among others).

### Placebo analysis

To ensure that our findings are not driven by model misspecification, we perform a placebo analysis, where the outcome variable is the demand for vaccination against tick-borne encephalitis (TBE). Since the meningococcal disease and TBE are unrelated diseases, we expect the news related to meningococcal meningitis to have little effect on the uptake of vaccinations against TBE.

The results reported in Table 6 indicate that 1–4 months after the release of the news, meningitis-related articles have moderate (up to 3%) and statistically insignificant positive effects on the uptake of vaccinations against TBE. The positive (albeit insignificant) effects might be due to the raising awareness of immunisation, the contact with the GP and the discussion of the available vaccinations as a result of the mass media coverage of meningococcal meningitis.

**Table 6:** Fixed effects models of uptake of vaccination against tick-borne encephalitis

	Coeff	S.E.
Number of meningitis-related county-specific articles		
Current	$-$1.307	[16.56]
1 Month lag	13.01	[25.29]
2 Months lag	1.295	[17.88]
3 Months lag	12.61	[11.22]
4 Months lag	22.42	[15.82]
IMD cases per 100,000 population aged 0–17		
Current	$-$1.370	[21.59]
1 Month lag	12.65	[28.17]
2 Months lag	26.37	[20.87]
3 Months lag	32.95*	[16.09]
4 Months lag	31.67	[27.98]
County effects	yes	
Monthly date effects	yes	
Mean of outcome	673	
Number of observations	1,200	

Robust standard errors in brackets, *** *p* < 0.01, ** *p* < 0.05, * *p* < 0.1

Sample: years 2014–2018

All meningitis-related articles are considered. The outcome is the monthly vaccination rate per 100,000 population aged 0–17

## Discussion

In this paper, we analysed how the uptake of vaccinations against the meningococcal disease responds to mass media coverage of the disease and to actual incidence rates. Using county-level monthly panel data from Hungary, we found evidence that the demand for all three types of meningococcal vaccination responds to mass media coverage, while little evidence is found that the actual incidence or mortality rate of invasive meningococcal disease influences vaccination demand.

Meningitis-related online articles have a positive effect on the demand for Men C, Men B and Men ACWY vaccinations. This is more than a timing effect, and the total effect remains positive over a 5-month horizon. The increasing demand in relative terms is the strongest for the least demanded type of vaccination (vaccination against serogroups A, C, W, Y), with an immediate (within 1 month) effect of approximately 114%, compared to the average rate of vaccination. The positive effect on Men C and Men ACWY vaccination demand clearly decreases over the analysed 5-month horizon. The impact is more persistent on the demand for Men B vaccination, suggesting that the media coverage raised awareness of the availability of this vaccination or modified its perceived costs or benefits even beyond a 5-month time horizon. Another possible reason for persistence is that parents of newborns need to wait until the age of 2 months to vaccinate. The results altogether imply that individuals are not perfectly informed about the severity or incidence of invasive meningococcal disease and/or that their vaccination decisions are not fully rational; otherwise, media coverage would not have an effect on vaccination demand.

We captured mass media coverage of the meningococcal disease with the coverage by the four most popular online journals in Hungary. Although this is a restricted set of the media outlets (newspapers, television and radio are excluded), we see that the four online journals typically reported the same meningitis-related cases.^11^ In the studied period, internet penetration increased from 53 percent to 83 percent [61], nevertheless, households without internet access may have observed less of the news reported in our database. As a result, we underestimate the effect of news on vaccinations – the effect would be likely bigger if we could restrict the sample to households whom the news reached.

Our paper contributes to the literature by documenting the strong influence of mass media coverage of a disease on vaccination uptake. The findings point to the responsibility of the mass media in influencing health-related decisions, specifically, decisions related to vaccination. Our findings are also particularly important in our era in which the reluctance or refusal to vaccinate is a global health threat [1]. As we see in the context of our paper, the media coverage of invasive meningococcal disease is rather unbalanced and selective, and the excessive coverage of a single death event caused by invasive meningococcal disease leads to a surge in vaccination rates. As Nyhan et al. [62] suggest, the content and design of information received by the population may matter for vaccination beliefs and intentions to vaccinate. Thus, to mitigate the influence of the selective and often misleading contents of the media, health specialists and policymakers should strive to provide objective and clear information related to the spread of diseases and vaccinations both in the mass media and other channels, such as information leaflets.

Some limitations of our study need to be mentioned. Our data are restricted to monthly county-level statistics of vaccination purchases and disease occurrence. Thus, we could not analyse heterogeneities in the response to mass media coverage by household level characteristics, nor could we conduct a refined analysis of the spatial patterns in the response of vaccination uptake to a new occurrence of the invasive meningococcal disease. In addition, due to our identification strategy, our estimates provide a lower bound for the total effect of mass media coverage on vaccination uptake. A further data limitation is that county-specific fatalities are observed only annually. Additionally, the impact of mass media was analysed on a disease with rapid progression and a high fatality rate. The analysis of the impact of mass media on the demand for vaccinations against diseases with different characteristics (with slower progression, such as the human papillomavirus, or with lower mortality rate, such as the rotavirus) remains to future research. It is also an important issue whether our results are externally valid to other countries. We believe that our results have relevance to other institutional settings where a vaccination is not mandatory; in such settings, the individuals’ decision on whether to vaccinate is strongly influenced by disease- or vaccination-related news in the mass media.

## Figures and Tables

**Fig. 7:**
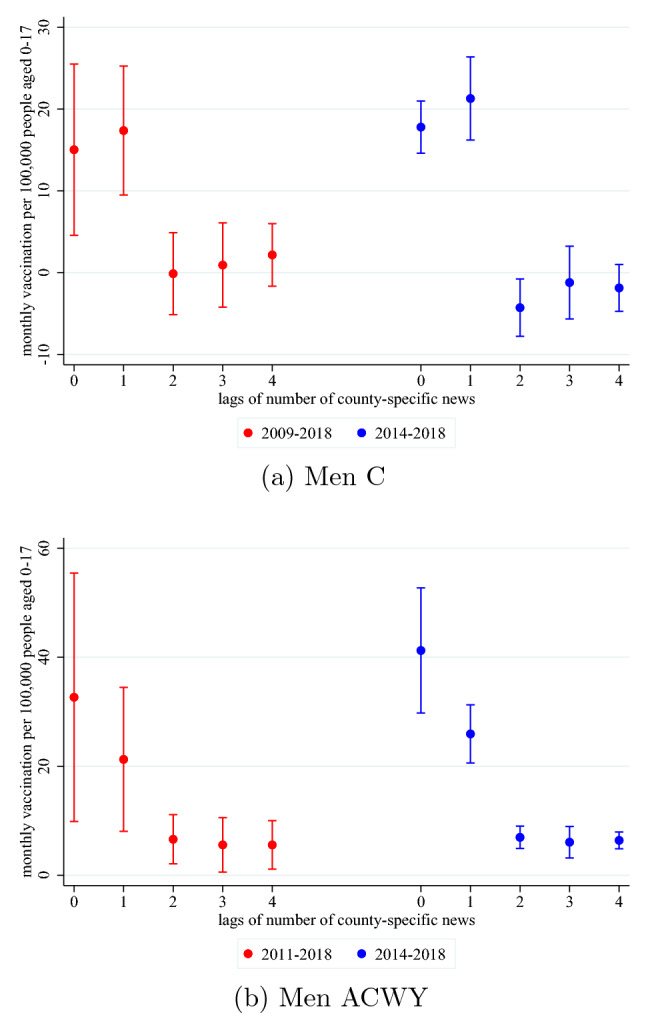
Estimated effects of meningitis-related online articles on vaccination (with 95% CI)

## A: Specification check: exclusion of fixed effects

**Table 7:** OLS and fixed effects models of vaccination uptake

	(1)	(2)	(3)	(4)	(5)	(6)
	Men C	Men B	Men ACWY	Men C	Men B	Men ACWY
Number of county-specific articles						
Current	58.42$$***$$	30.87$$***$$	50.54$$***$$	26.69$$***$$	20.78$$***$$	47.13$$***$$
	[8.527]	[2.876]	[5.481]	[8.814]	[2.852]	[5.677]
1 Month lag	96.26$$***$$	25.68$$***$$	34.16$$***$$	67.05$$***$$	16.31$$***$$	31.04$$***$$
	[7.364]	[2.040]	[2.723]	[6.412]	[1.555]	[2.329]
2 Months lag	46.97$$***$$	31.00$$***$$	12.59$$***$$	17.24$$**$$	21.54$$***$$	9.437$$***$$
	[6.887]	[3.555]	[1.061]	[6.825]	[3.027]	[0.605]
3 Months lag	50.65$$***$$	56.62$$***$$	11.23$$***$$	21.12$$***$$	47.23$$***$$	8.091$$***$$
	[4.692]	[5.798]	[1.216]	[3.821]	[6.671]	[1.256]
4 Months lag	46.62$$***$$	23.68$$***$$	12.10$$***$$	14.30$$***$$	13.24$$***$$	8.739$$***$$
	[10.22]	[2.956]	[1.412]	[4.671]	[1.995]	[0.5217]
IMD cases per 100,000 population aged 0–17						
Current	$-$13.85	$-$6.711	$-$3.655	$-$17.04	$-$9.371	$-$3.192
Population aged 0–17	[16.45]	[5.767]	[2.950]	[12.30]	[6.397]	[3.004]
1 Month lag	$$-$$12.96	$$-$$10.85$$**$$	$$-$$3.032	$$-$$14.25	$$-$$11.80$$***$$	$$-$$2.337
Population aged 0–17	[16.13]	[4.434]	[2.644]	[11.38]	[3.276]	[1.813]
2 Months lag	$-$10.38	$-$6.504	0.0307	$-$10.09	$-$7.229$*$	0.707
Population aged 0–17	[17.48]	[5.575]	[2.560]	[9.365]	[3.670]	[1.793]
3 Months lag	$$-$$14.48	$$-$$10.71$$**$$	$$-$$0.705	$$-$$12.94	$$-$$11.65$$*$$	0.0894
Population aged 0–17	[15.64]	[5.004]	[1.799]	[8.163]	[6.352]	[1.382]
4 Months lag	$$-$$21.64	$$-$$10.50$$*$$	$$-$$1.824	$$-$$19.78$$**$$	$$-$$10.84$$***$$	$$-$$0.894
Population aged 0–17	[14.23]	[5.496]	[1.764]	[7.318]	[3.610]	[1.379]
County effects	No	no	no	yes	yes	yes
Monthly date effects	No	no	no	no	no	no
Mean of outcome	680	72	36	680	72	36
Number of observations	1200	1080	1200	1200	1080	1200

Robust standard errors in brackets, $$***$$*p* < 0.01, $$**$$ *p* < 0.05, $$*$$ *p* < 0.1. Sample: years 2014–2018 (from July 2014 in case of Men B). All meningitis-related articles are considered in models (1–6). The outcome is the monthly vaccination rate per 100,000 population aged 0–17

## B: Heterogeneity by time

We re-estimate equation (3) on the entire range of years for which we have data and the analysed vaccinations were available, i.e. years 2009–2018 for the Men C vaccination, and years 2011–2018 for the Men ACWY vaccination. (Men B vaccination became available in year 2014, at which year our sample period begins in the baseline specifications).

Although the point estimates are qualitatively robust to the sample period, the estimates are more precise over years 2014–2018. The increased precision is due to the fact that most of the meningitis-related articles were published in years 2016-2017, and internet access of households increased gradually throughout 2009–2018, from 53% in 2009 to 83% in 2018 (source: [61]). Therefore, our estimated results mostly originate from the vaccination response to online media coverage in the second half of the entire observation period, the observations from the earlier years mostly add a noise to the estimates (Fig. 7).

## Footnotes

1 Neisseria meningitidis.

2 Through droplets of respiratory or throat secretions from carriers.

3 Such as BCG, MMR, DPTa-IPV-HiB, PCV13.

4 We calculated the unit price and subsidisation rate based on the vaccination statistics provided by the National Health Insurance Management. See Sect. 4 for further details.

5 These data are available in the secure data room of the Hungarian Central Statistical Office and the Centre for Economic and Regional Studies.

6 The four journals are 24.hu , blikk.hu, index.hu and origo.hu.

7 Although we used the keyword meningitis (and not meningococcal disease, meningococcal meningitis or meningococcal sepsis, which would be medically more precise), all the articles that include the keyword meningitis refer in fact either to the meningococcal disease or to meningococcal vaccinations. The medical term meningococcus does not appear in article titles in the mass media.

8 The Stata code of the regressions is available at https://github.com/szabomorvai/vaccinations.git.

9 We do not observe the age of patients buying the vaccinations. However, we know that IMD mostly affects young children and adolescents. Therefore, we assume that mainly these cohorts are vaccinated; hence we relate the vaccination purchases to the population size of ages 0–17. The age cut-off is also set at age 17 because the T-STAR database provides county-specific population size only for certain age intervals, and the calculation of the age 0–17 population size is feasible based on these statistics.

10 $$Corr(article,IMD) = 0.084$$ and $$Corr(death,IMD) = 0.058$$.

11 As a check for representativeness, we explored the evening news archives of RTL Klub (https://rtl.hu/), one of the largest national commercial TV channels) and found that there are less reported meningitis cases compared to the online journals, and all mentioned cases can be found in our news database. As a further check, we examined two local newspapers (Hajdú-Bihari Napló in a library archive and Kelet-Magyarország at https://library.hungaricana.hu/hu/collection/helyi_lapok_nyiregyhaza_keletmagyarorszag/) for two random years within the period of the study. We have not found any mentions of meningitis cases whatsoever.
